# Implication of EEG theta/alpha and theta/beta ratio in Alzheimer’s and Lewy body disease

**DOI:** 10.1038/s41598-022-21951-5

**Published:** 2022-11-04

**Authors:** Kyoungwon Baik, Jin Ho Jung, Seong Ho Jeong, Seok Jong Chung, Han Soo Yoo, Phil Hyu Lee, Young H. Sohn, Seung Wan Kang, Byoung Seok Ye

**Affiliations:** 1 Department of Neurology, Yonsei University College of Medicine, 50-1 Yonsei-ro, Seodaemun-gu, Seoul, 03722 South Korea; 2 iMediSync Inc., Seoul, Korea; 3 Data Center for Korean EEG, College of Nursing, Seoul National University, Seoul, Korea

**Keywords:** Neuroscience, Neurology

## Abstract

We evaluated the patterns of quantitative electroencephalography (EEG) in patients with Alzheimer’s disease (AD), Lewy body disease (LBD), and mixed disease. Sixteen patients with AD, 38 with LBD, 20 with mixed disease, and 17 control participants were recruited and underwent EEG. The theta/alpha ratio and theta/beta ratio were measured. The relationship of the log-transformed theta/alpha ratio (TAR) and theta/beta ratio (TBR) with the disease group, the presence of AD and LBD, and clinical symptoms were evaluated. Participants in the LBD and mixed disease groups had higher TBR in all lobes except for occipital lobe than those in the control group. The presence of LBD was independently associated with higher TBR in all lobes and higher central and parietal TAR, while the presence of AD was not. Among cognitively impaired patients, higher TAR was associated with the language, memory, and visuospatial dysfunction, while higher TBR was associated with the memory and frontal/executive dysfunction. Increased TBR in all lobar regions and temporal TAR were associated with the hallucinations, while cognitive fluctuations and the severity of Parkinsonism were not. Increased TBR could be a biomarker for LBD, independent of AD, while the presence of mixed disease could be reflected as increased TAR.

## Introduction

Alzheimer’s disease (AD) and Lewy body disease (LBD) account for the majority of degenerative dementia cases. AD is the most common cause of degenerative dementia characterized by progressive memory decline and cognitive dysfunction associated with the amyloid plaques and neurofibrillary tangles^1^. LBD, the second most common cause of degenerative dementia, encompasses Parkinson’s disease (PD), and dementia with Lewy bodies (DLB). The pathological hallmark of LBDs is the presence of Lewy bodies related to alpha-synuclein^2,3^. They share the similar clinical characteristics, including REM sleep behavior disorder, parkinsonism, fluctuating cognition, and visual hallucinations. An accurate diagnosis of the etiology of dementia is important for appropriate clinical management and the development of disease-modifying treatments. Although in vivo amyloid positron emission tomography (PET) and dopamine transporter (DAT) imaging have enabled the antemortem diagnosis of AD and LBD^4,5^, performing DAT imaging as a routine evaluation in the memory clinic could be difficult due to cost-related issues. In addition, the diagnostic sensitivity of LBD is suboptimal especially in patients without visual hallucinations and cognitive fluctuation, and in those with mixed AD pathology^6^. Considering that the co-occurrence of AD and LBD in patients with cognitive impairment is not uncommon^7,8^, biomarkers that sensitively detect LBD even with concomitant AD pathology could be helpful in enhancing its diagnostic accuracy.

Electroencephalography (EEG) is a promising noninvasive and cost-effective biomarker for the diagnosis of dementia. Abnormal functional and structural connectivity have been consistently identified^9^ in dementia. EEG could detect these changes sensitively since it provides information on the cortical neuronal synchronization, and functional and effective brain connectivity^10^. In addition, cholinergic dysfunction in AD^11^ and LBD^12^ could affect the dominant background rhythm^13^ and fast cortical activity^14^.

EEG is easily accessible to track the severity of cognitive dysfunction in degenerative diseases^15,16^, and predicts the progression to dementia^17^. Previously, various EEG analytic methods have been used to characterize AD and LBD, including visual rating^17–19^, quantitative methods using coherence^20,21^, power^17,19–22^, power ratio^17,23–28^, and dominant frequency^22,29^, mathematical classifier^30,31^, and connectivity analyses^18^. Several findings of EEG abnormalities associated with AD have been reported; increased posterior EEG slowing^18,31^, increased theta power in temporal, parietal and occipital lobes^22^, decreased posterior alpha power and higher occipital or widespread delta power^23,24^, increased delta and theta power with decline of fast wave activities^25^, reduced temporal lobe alpha coherence^20,21^ and interhemispheric theta coherence^21^. EEG could be used as classification of AD patients from control participants with various but relatively good sensitivity (70–89%) and specificity (77–90%)^21,23,25,27,30–32^. In addition, EEG biomarkers could predict the conversion from mild cognitive impairment (MCI) to AD; alpha and theta power and mean frequency from T5-O1 derivation, activity in the beta frequency range^33^, increased high alpha frequency^34^. However, DLB patients^17,19,23,24,26,30,35–37^ and even those with PD^24,26^ have more prominent EEG changes than AD patients, and EEG is regarded as a supportive biomarker for the diagnosis of DLB^38^. As patients with pathologically confirmed mixed disease with AD and DLB could have various clinical diagnoses of AD, DLB, and PD dementia^39^, consideration for mixed disease is needed to find the true association of EEG abnormalities with AD and LBD. To the best of our knowledge, only one study has compared the EEG features of DLB with AD, DLB without AD, and pure AD patients^19^. However, the independent effects of AD and LBD on EEG features have not yet been evaluated.

In this study, we investigated the effects of AD and LBD on the patterns of spectral power computation of EEG acquired from a low-density scalp EEG in amyloid PET-confirmed AD and DAT PET-confirmed LBD patients whose diagnoses were based on meticulous clinical evaluation and supported by^18^F-fluorodeoxyglucose (FDG) PET^8,40^. The theta/alpha ratio has been proposed as an easy-to-use quantitative EEG (QEEG) biomarker for differentiating AD patients from control participants^25,27^. However, LBD patients have higher theta/alpha ratio than control participants^26^ and even patients with AD^17,19,26^. Previous studies also showed that patients with MCI due to LBD have decreased beta power and increased theta power than control subjects^41,42^. Also, decreased beta power^17,41^ and increased theta^17^ or pre-alpha^41^ power was reported in MCI due to LBD group compared with MCI due to AD group, while the two groups had comparable alpha power^41^. Based on these previous studies, we hypothesized that theta/beta ratio could be a better index for detecting LBD than theta/alpha ratio, independent of AD.

## Results

### Demographic and clinical characteristics

The demographic and clinical characteristics of the participants are presented in Table 1. There were no significant differences in age, sex, and education among the AD, LBD, mixed disease, and control groups. The three disease groups had lower mean Korean version of the Mini-Mental State Examination (K-MMSE) scores than the control group (p = 0.021). There were no significant differences in age, sex, education, disease duration, K-MMSE score, and Clinical Dementia Rating Sum of Boxes (CDR-SOB) among the three disease groups. The mixed disease and LBD groups had higher mean Unified Parkinson’s Disease Rating Scale (UPDRS) part III score than the AD group (p < 0.001). In addition, patients in the LBD group had higher UPDRS part III score than those in the mixed disease group. The number of patients with cognitive fluctuation (p < 0.001), hallucinations (p = 0.028), and RBD (p = 0.006) were higher in the mixed disease and LBD groups than in the AD group. The LBD group had a higher number of patients with parkinsonism and fluctuation than the mixed disease group. There were significant differences in the frequency of acetylcholinesterase inhibitor (p < 0.001) and antidepressant usage (p = 0.001) among control, AD, LBD, and mixed disease groups.

**Table 1:** Demographics and clinical characteristic.

Group	Control	AD	Mixed disease	LBD	P value^1^	P value^2^
Number	17	16	20	38		
Age	73.4 (3.3)	75.6 (9.0)	79.3 (5.9)	77.1 (7.3)	0.063	0.315
Sex, men	10 (58.8)	4 (25.0)	9 (45.0)	22 (57.9)	0.129	0.084
Education	11.4 (2.8)	7.9 (4.0)	9.0 (5.0)	9.2 (5.7)	0.224	0.713
Duration	NA	3.4 (2.4)	4.6 (2.8)	3.2 (2.3)	NA	0.131
K-MMSE	28.3 (2.2)	18.9 (4.6)	17.1 (5.7)	19.3 (7.5)	0.021	0.455
CDR-SOB	NA	3.7 (2.7)	5.6 (2.2)	4.2 (3.8)	NA	0.251
UPDRS part III	NA	14.5 (7.7) ^b,c^	24.5 (10.9) ^b,d^	31.9 (13.7)^c,d^	NA	< 0.001
Parkinsonism, no	NA	5 (31.3)^b,c^	15 (75.0)^b,d^	37 (97.4)^c,d^	NA	< 0.001
Fluctuation, no	NA	0 (0.0)^b,c^	6 (30.0)^b,d^	25 (65.8)^c,d^	NA	< 0.001
Hallucination, no	NA	0 (0.0)^b,c^	6 (30.0)^b^	12 (31.6)^c^	NA	0.028
RBD, no	NA	0 (0.0)^b,c^	7 (35.0)^b^	17 (44.7)^c^	NA	0.006
AChEI, no	0	2 (12.5)^b^	10 (50.0)^a,b^	14 (36.8)^a^	0.001	0.061
Antidepressant, no	0	1 (6.3)	7 (35.0)^a^	8 (21.1)^a^	0.017	0.110
Antipsychotic, no	0	1 (6.3)	1 (5.0)	3 (7.9)	0.856	1.000
Benzodiazepine, no	0	1 (6.3)	4 (20.0)	6 (15.8)	0.215	0.569
Dopaminergic, no	0	0 (0.0)	2 (10.0)	3 (7.9)	0.499	0.595

Data are expressed in mean (SD) or number (percentage). Data are results of chi-square tests or analyses of variance as appropriate.

*AChEI* acetylcholinesterase inhibitor, *AD* Alzheimer’s disease, *CDR-SOB* clinical dementia rating sum of boxes, *K-MMSE* Korean version of Mini-Mental State Examination, *LBD* Lewy body disease, *SD* standard deviation, *UPDRS* unified Parkinson’s disease rating scale.

^1^P values are results of comparisons between all four study groups.

^2^P values are results of comparisons between the three disease groups.

^a^Significantly different in the comparison with the control group.

^b^Significantly different in the comparison between the AD and mixed disease groups.

^c^Significantly different in the comparison between the AD and pure LBD groups.

^d^Significantly different in the comparison between the mixed disease and LBD groups.

### Groupwise comparisons of mean lobar TAR and TBR

**Figure 1:**
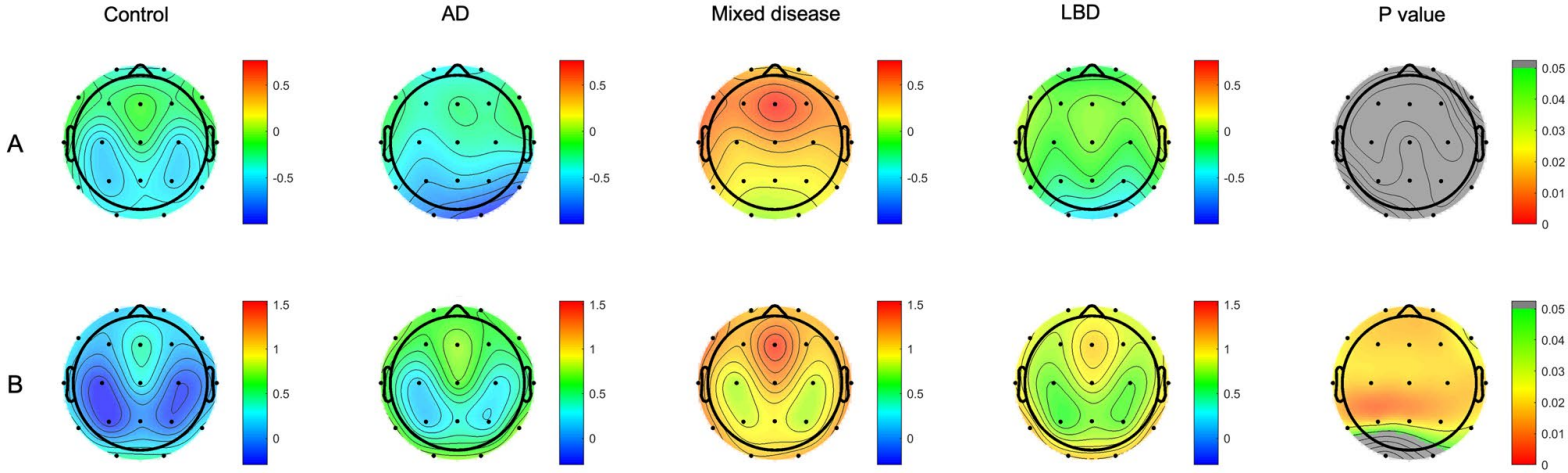
TAR (**A**) and TBR (**B**) between groups. P values are results of general linear models for TAR and TBR using the disease group as a predictor after controlling for age, sex, education, use of AchEI and antidepressants. P values are FDR corrected values. *AChEI* acetylcholinesterase inhibitor, *AD* Alzheimer’s disease, *LBD* Lewy body disease, *FDR* false discovery rate, *TAR* log transformed theta/alpha ratio, *TBR* log transformed theta/beta ratio.

There was no significant group difference in the log-transformed theta/alpha ratio (TAR) (Table2, Fig. 1A). General linear model (GLM) revealed that patients in the mixed disease and LBD groups had higher mean log-transformed theta/beta ratio (TBR) than those in the control group in the frontal, central, temporal, and parietal lobes (Table 2, Fig. 1B). Relative band power analysis in each frequency revealed that the three disease groups had higher theta band power than the control group in all lobar regions. The mixed disease group had higher theta band power than the AD group in the frontal, central, temporal, and parietal lobes, and the mixed disease group had higher theta band power than the LBD group in the frontal lobe (Supplementary Table S1). The three disease groups had lower alpha1 band power in the frontal and temporal lobes than the control group. The mixed disease and LBD groups had lower occipital alpha1 band power than the control group, and the mixed disease group had lower occipital alpha1 band power than the AD group. There were no significant group differences in the other frequency band power.

**Table 2:** Group-wise comparisons of mean lobar TAR and TBR.

Model 1	Control	AD	Mixed disease	LBD	P value
**TAR**					
Frontal TAR	− 0.20 (0.85)	− 0.27 (0.36)	0.53 (0.60)	− 0.04 (0.97)	0.067
Central TAR	− 0.39 (0.80)	− 0.36 (0.59)	0.38 (0.62)	− 0.08 (0.88)	0.067
Temporal TAR	− 0.22 (0.85)	− 0.39 (0.57)	0.43 (0.69)	0.01 (1.01)	0.119
Parietal TAR	− 0.50 (0.67)	− 0.57 (0.66)	0.19 (0.60)	− 0.23 (0.88)	0.067
Occipital TAR	− 0.40 (1.29)	− 0.85 (0.83)	0.09 (0.82)	− 0.47 (1.11)	0.131
**TBR**					
Frontal TBR	0.23 (0.50)	0.68 (0.71)	1.22 (0.92)^a^	0.92 (0.99)^a^	0.030
Central TBR	− 0.02 (0.54)	0.41 (0.69)	0.98 (0.92)a	0.78 (0.93)a	0.020
Temporal TBR	0.24 (0.59)	0.71 (0.83)	1.28 (0.98)^a^	1.07 (1.12)^a^	0.030
Parietal TBR	− 0.14 (0.42)	0.27 (0.73)	0.90 (0.95)^a^	0.70 (1.03)^a^	0.030
Occipital TBR	0.41 (0.73)	0.74 (0.79)	1.14 (1.09)	1.07 (1.07)	0.114

Model 2	Control	ADCI	Mixed disease	LBD	P value
**TAR**					
Frontal TAR	− 0.20 (0.85)	− 0.27 (0.64)^b^	0.59 (0.64)^a,b,d^	− 0.15 (0.94)^d^	0.020
Central TAR	− 0.39 (0.80)	− 0.36 (0.59)^b^	0.42 (0.63)^a,b,d^	− 0.16 (0.86)^d^	0.025
Temporal TAR	− 0.22 (0.85)	− 0.39 (0.57)^b^	0.54 (0.73)^a,b,d^	− 0.12 (0.97)^d^	0.031
Parietal TAR	− 0.50 (0.67)	− 0.57 (0.66)^b^	0.24 (0.62)^a,b,d^	− 0.32 (0.86)^d^	0.031
Occipital TAR	− 0.40 (1.29)	− 0.85 (0.83)^b^	0.18 (0.86)^b,d^	− 0.60 (1.06)^d^	0.038
**TBR**					
Frontal TBR	0.23 (0.50)	0.68 (0.71)^b^	1.34 (0.83)^a,b,d^	0.80 (1.01)^a,d^	0.007
Central TBR	− 0.02 (0.54)	0.41 (0.69)^b^	1.06 (0.84)^a,b^	0.70 (0.96)^a^	0.007
Temporal TBR	0.24 (0.59)	0.71 (0.83)^b^	1.45 (0.90)^a,b^	0.91 (1.14)^a^	0.008
Parietal TBR	− 0.14 (0.42)	0.27 (0.73)^b^	1.01 (0.83)^a,b^	0.61 (1.08)^a^	0.007
Occipital TBR	0.41 (0.73)	0.74 (0.79)	1.34 (1.00)^a^	0.91 (1.10)^a^	0.038

Data are expressed in mean (standard deviation). P values are results of general linear models for TAR and TBR using the disease group as a predictor after controlling for age, sex, education, use of AchEI and antidepressants. P values are FDR corrected values. Model 1 was based on the disease categorization according to the presence of AD and LBD, while model 2 was based on the categorization according to the presence of ADCI (amyloid-positivity) and LBD.

*AChEI* acetylcholinesterase inhibitor, *AD* Alzheimer’s disease, *ADCI* Alzheimer’s disease-related cognitive impairment, *LBD* Lewy body disease, *FDR* false discovery rate, *TAR* log transformed theta/alpha ratio, *TBR* log transformed theta/beta ratio.

^a^Significantly different in the comparison with the control group.

^b^Significantly different in the comparison between the AD (or ADCI) and mixed disease groups.

^c^Significantly different in the comparison between the AD (or ADCI) and LBD groups.

^d^Significantly different in the comparison between the mixed disease and LBD groups.

### Effects of AD and LBD on TAR and TBR

Since the interaction effects of AD and LBD on TAR and TBR were not statistically significant in any lobar regions, the interaction term was removed from the statistical model (Table 3). The presence of LBD was associated with increased central and parietal TAR (false discovery rate (FDR)-corrected p < 0.05), but the presence of AD was not associated with TAR in any lobar region. The presence of LBD was associated with increased TBR in all lobar regions (FDR-corrected p < 0.05), while the presence of AD was not. Relative band power analysis in each frequency revealed that the effect of LBD was significant on theta power in all lobes, alpha1 power in the frontal, central, temporal, and occipital lobes, and beta3 power in the parietal lobe. The effect of AD was significant on theta power in the frontal and central lobes (Supplementary Table S2).

**Table 3:** Independent effects of AD (or ADCI) and LBD on lobar TAR and TBR.

Model 1	LBD effect		AD effect		LBD*AD	
	Beta (SE)	P value	Beta (SE)	P value	Beta (SE)	P value
**TAR**						
Frontal TAR	0.427 (0.208)	0.050	0.353 (0.190)	0.170	NA	NA
Central TAR	0.461 (0.190)	0.033	0.339 (0.174)	0.170	NA	NA
Temporal TAR	0.427 (0.210)	0.050	0.209 (0.192)	0.333	NA	NA
Parietal TAR	0.439 (0.187)	0.033	0.278 (0.171)	0.173	NA	NA
Occipital TAR	0.362 (0.264)	0.173	0.226 (0.241)	0.352	NA	NA
**TBR**						
Frontal TBR	0.669 (0.214)	0.005	0.393 (0.195)	0.170	NA	NA
Central TBR	0.757 (0.208)	0.005	0.352 (0.190)	0.170	NA	NA
Temporal TBR	0.742 (0.236)	0.005	0.338 (0.216)	0.173	NA	NA
Parietal TBR	0.791 (0.221)	0.005	0.326 (0.202)	0.173	NA	NA
Occipital TBR	0.554 (0.239)	0.033	0.228 (0.219)	0.333	NA	NA

Model 2	LBD effect		ADCI effect		LBD*ADCI	
	Beta (SE)	P value	Beta (SE)	P value	Beta (SE)	P value
**TAR**						
Frontal TAR	0.435 (0.205)	0.037	0.432 (0.190)	0.045	NA	NA
Central TAR	0.463 (0.188)	0.024	0.386 (0.174)	0.045	NA	NA
Temporal TAR	0.445 (0.208)	0.037	0.320 (0.193)	0.101	NA	NA
Parietal TAR	0.441 (0.186)	0.026	0.319 (0.172)	0.077	NA	NA
Occipital TAR	1.00 (0.37)	0.009	0.77 (0.30)	0.013	1.10 (0.48)	0.024
**TBR**						
Frontal TBR	0.691 (0.208)	0.002	0.547 (0.193)	0.045	NA	NA
Central TBR	0.773 (0.204)	0.002	0.468 (0.189)	0.045	NA	NA
Temporal TBR	0.776 (0.229)	0.002	0.545 (0.213)	0.045	NA	NA
Parietal TBR	0.811 (0.217)	0.002	0.462 (0.201)	0.045	NA	NA
Occipital TBR	0.591 (0.234)	0.023	0.439 (0.217)	0.059	NA	NA

Data are results of general linear models for TAR and TBR using the presence of AD and LBD as predictors after controlling for age, sex, education, use of AchEI antidepressants. Model 1 was based on the disease categorization according to the presence of AD and LBD, while model 2 was based on the categorization according to the presence of ADCI (amyloid-positivity) and LBD. P values are FDR corrected values.

*AChEI* acetylcholinesterase inhibitor, *AD* Alzheimer’s disease, *ADCI* Alzheimer’s disease-related cognitive impairment, *FDR* false discovery rate, *LBD* Lewy body disease, *SE* standard error, *TAR* log transformed theta/alpha ratio, *TBR* log transformed theta/beta ratio.

### Sensitivity analyses based on different disease classifications

To determine the effects of different classifications based on the presence of AD-related cognitive impairment (ADCI) (rather than typical AD) or that based on the presence of DLB (rather than LBD), further GLMs were performed with two different group classifications. First, a new classification based on the presence of ADCI and LBD revealed that the mixed disease (with both ADCI and LBD) group had higher TAR than the ADCI and LBD groups in all lobar regions, and higher frontal, central, temporal, and parietal TAR than the control group (Table 2). In addition, the mixed disease and LBD groups had higher TBR than the control group in all lobar regions. Also, the mixed disease group had higher frontal TBR than the ADCI and LBD groups, and higher central, temporal, and parietal TBR than the ADCI group. When the independent and interaction effects of ADCI and LBD were investigated, there was a significant ADCI*LBD interaction effect on occipital TAR (Table 3). The presence of LBD was associated with increased TAR and TBR in all lobar regions. Additionally, the presence of ADCI was associated with increased TAR in the frontal, central and occipital lobes, and increased TBR in the frontal, central, temporal, and parietal lobes.

Second, another new classification based on the presence of AD and DLB revealed that the AD, mixed disease, and DLB groups had higher frontal, central, temporal, and parietal TBR than the control group (Supplementary Table S3). Additionally, the mixed disease group had higher frontal, central, and parietal TBR than the AD group. When the independent and interaction effects of AD and DLB were investigated, there was no significant interaction effect (Supplementary Table S4). The presence of DLB was associated with increased TBR in all lobar regions, but the presence of DLB was not associated with TAR in any lobar region. In addition, the presence of AD was marginally associated with increased frontal, central, temporal, and parietal TBRs (FDR-corrected p = 0.05 for frontal a nd central TBR, FDR-corrected p = 0.055 for temporal and parietal TBR).

### Association of TAR and TBR with clinical features

Logistic regression analyses revealed that higher TBR in all regions and temporal TAR were associated with an increased risk of hallucination (FDR-corrected p < 0.05) (Table 4). In contrast, the risk of fluctuation was not significantly associated with TAR or TBR.

**Table 4:** Association of TAR and TBR with the presence of clinical symptoms.

	Hallucination		Fluctuation	
	OR (95% CI)	P value	OR (95% CI)	P value
**TAR**				
Frontal TAR	2.79 (1.12–6.97)	0.080	0.78 (0.42–1.45)	0.610
Central TAR	2.90 (1.06–7.94)	0.082	0.73 (0.30–1.44)	0.610
Temporal TAR	3.55 (1.35–9.35)	0.047	0.77 (0.41–1.43)	0.610
Parietal TAR	2.61 (1.04–6.54)	0.082	0.89 (0.46–1.71)	0.852
Occipital TAR	2.22 (1.06–4.67)	0.082	0.79 (0.46–1.36)	0.610
**TBR**				
Frontal TBR	3.47 (1.39–8.67)	0.047	0.92 (0.53–1.60)	0.971
Central TBR	3.27 (1.27–8.39)	0.047	1.01 (0.57–1.78)	0.976
Temporal TBR	2.91 (1.24–6.82)	0.047	0.91 (0.54–1.51)	0.852
Parietal TBR	3.55 (1.44–8.73)	0.047	0.97 (0.58–1.64)	0.971
Occipital TBR	3.47 (1.34–9.01)	0.047	0.85 (0.51–1.43)	0.716

Data are results of logistic regression analyses for the presence of clinical symptoms performed in cognitively impaired patients using age, sex, education, disease duration, use of AchEI and antidepressants. P values are FDR corrected values.

*AChEI* acetylcholinesterase inhibitor, *CI* confidence interval, *FDR* false discovery rate, *TAR* log transformed theta/alpha ratio, *TBR* log transformed theta/beta ratio, *OR* odds ratio, *UPDRS* Unified Parkinson’s disease rating scale.

### Association of TAR and TBR with neuropsychological test and UPDRS part III scores

GLM for neuropsychological test scores revealed that increased TAR in all lobes were associated with lower memory and visuospatial domain scores (FDR-corrected p < 0.05), increased temporal and occipital TAR were associated with the lower language domain score. Increased TBR in temporal and parietal lobes were associated with the lower memory domain score and increased TBR in the parietal lobe was associated with lower frontal/executive score (Table 5). GLM for the UPDRS part III score revealed that UPDRS part III score was positively associated with central, parietal, and occipital TBR, however, this association was not statistically significant after FDR correction (Supplementary Table S5).

**Table 5:** Association of TAR and TBR with neuropsychological test scores.

	Frontal TAR		Central TAR		Temporal TAR		Parietal TAR		Occipital TAR	
	Beta (SE)	P value	Beta (SE)	P value	Beta (SE)	P value	Beta (SE)	P value	Beta (SE)	P value
Attention	− 0.06 (0.16)	0.754	− 0.10 (0.17)	0.603	− 0.10 (0.15)	0.559	− 0.06 (0.17)	0.756	0.02 (0.13)	0.894
Language	− 0.44 (0.21)	0.081	− 0.53 (0.23)	0.060	− 0.52 (0.20)	0.047	− 0.54 (0.23)	0.060	− 0.44 (0.17)	0.047
Memory	− 0.27 (0.10)	0.047	− 0.29 (0.11)	0.047	− 0.27 (0.10)	0.047	− 0.30 (0.11)	0.047	− 0.21 (0.08)	0.047
Frontal/executive	− 0.21 (0.15)	0.194	− 0.26 (0.16)	0.157	− 0.32 (0.14)	0.060	− 0.29 (0.16)	0.131	− 0.21 (0.12)	0.136
Visuospatial	− 1.22 (0.46)	0.047	− 1.33 (0.51)	0.047	− 1.27 (0.45)	0.047	− 0.15 (0.50)	0.047	− 1.01 (0.39)	0.047

	Frontal TBR		Central TBR		Temporal TBR		Parietal TBR		Occipital TBR	
	Beta (SE)	P value	Beta (SE)	P value	Beta (SE)	P value	Beta (SE)	P value	Beta (SE)	P value
Attention	− 0.18 (0.15)	0.264	− 0.25 (0.15)	0.136	− 0.14 (0.13)	0.343	− 0.24 (0.14)	0.136	− 0.13 (0.13)	0.359
Language	− 0.24 (0.21)	0.279	− 0.30 (0.21)	0.194	− 0.29 (0.18)	0.154	− 0.29 (0.20)	0.192	− 0.28 (0.18)	0.164
Memory	− 0.22 (0.10)	0.060	− 0.22 (0.10)	0.060	− 0.22 (0.09)	0.047	− 0.23 (0.09)	0.047	− 0.17 (0.09)	0.091
Frontal/executive	− 0.27 (0.14)	0.093	− 0.34 (0.14)	0.053	− 0.28 (0.12)	0.058	− 0.35 (0.13)	0.047	− 0.25 (0.12)	0.079
Visuospatial	− 0.81 (0.45)	0.127	− 1.03 (0.46)	0.060	− 0.85 (0.40)	0.070	− 1.01 (0.43)	0.058	− 0.72 (0.40)	0.127

Data are results of general linear models for neuropsychological test scores performed in cognitively impaired patients using sex, disease duration, use of AchEI antidepressants as covariates. P values are FDR corrected values.

*AChEI* acetylcholinesterase inhibitor, *FDR* false discovery rate, *TAR* log transformed theta/alpha ratio, *TBR* log transformed theta/beta ratio, *SE* standard error.

### Diagnostic performance of TAR and TBR

**Figure 2:**
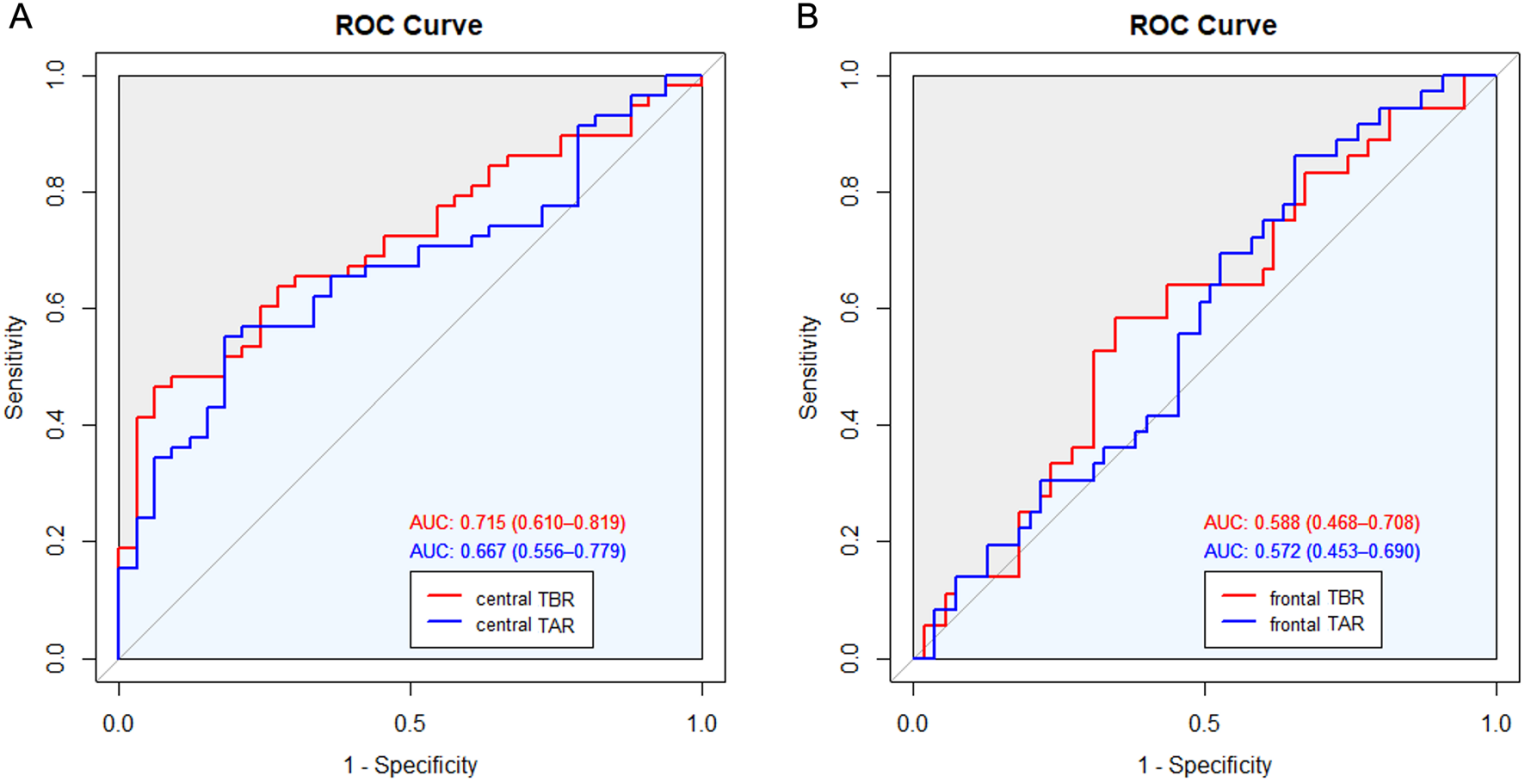
ROC curves predicting the presence of LBD based on central TAR and central TBR (**A**). ROC curves predicting the presence of AD based on frontal TAR and frontal TBR (**B**). *AD* Alzheimer’s disease, *AUC* area under the curve, *ROC* receiver operating characteristic, *TAR* log-transformed theta/alpha ratio, *TBR* log-transformed theta/beta ratio.

ROC curve analyses for the presence of LBD revealed that central TBR (AUC = 0.72, 95% CI 0.61–0.82), and parietal TBR (AUC = 0.72, 95% CI 0.61–0.82) revealed the highest AUC, followed by frontal TBR (AUC = 0.68, 95% CI 0.57–0.79), temporal TBR (AUC = 0.68, 95% CI 0.57–0.79), central TAR (AUC = 0.67, 95% CI 0.56–0.78), parietal TAR (AUC = 0.66, 95% CI 0.55–0.77), occipital TBR (AUC = 0.65, 95% CI 0.54–0.76), frontal TAR (AUC = 0.65, 95% CI 0.54–0.76), temporal TAR (AUC = 0.65, 95% CI 0.53–0.76), and occipital TAR (AUC = 0.59, 95% CI 0.47–0.71). Although central TBR revealed the highest AUC value, there was no significant difference between the AUC based on central TBR and that based on central TAR (P = 0.345) (Fig. 2A). ROC curve analyses for the presence of AD revealed that frontal TBR (AUC = 0.59, 95% CI 0.47–0.71) revealed the highest AUC, followed by frontal TAR (AUC = 0.57, 95% CI 0.45–0.69), parietal TAR (AUC = 0.57, 95% CI 0.41–0.65), central TBR (AUC = 0.59, 95% CI 0.45–0.69), temporal TBR (ACU = 0.57, 95% CI 0.45–0.68), central TAR (AUC = 0.56, 95% CI 0.45–0.68), parietal TBR (AUC = 0.56, 95% CI 0.44–0.68), temporal TAR (AUC = 0.54, 95% CI 0.42–0.66), occipital TAR (AUC = 0.53, 95% CI 0.41–0.65), occipital TBR (AUC = 0.53, 95% CI 0.40–0.65). Although frontal TBR revealed the highest AUC value, there was no significant difference between the AUC based on frontal TBR and that based on frontal TAR (P = 0.728) (Fig. 2B).

## Discussion

In this study, we evaluated the patterns of TBR and TAR of QEEG in patients with AD and/or LBD and their relationship with clinical features and cognitive dysfunction. Our major findings were as follows. First, LBD, but not AD, was independently associated with higher TBR, especially TBR in the central and parietal had the highest accuracy in predicting the presence of LBD. Second, the presence of AD and that of ADCI (amyloid-positivity) had different effects on TAR, which was mainly driven by prominently increased TAR in the mixed disease (ADCI with LBD) group. Third, in patients with cognitive impairment, increased TBR in all lobar regions and temporal TAR were associated with an increased risk of hallucinations, while cognitive fluctuations and the severity of parkinsonism were not associated with TAR or TBR. Fourth, increased TAR was associated with language, memory and visuospatial dysfunction, while increased TBR was associated with memory and frontal/executive dysfunction. Taken together, our findings suggest that an increased TBR, especially in the central and parietal lobes, was related to LBD, and simultaneously increased TAR and TBR could suggest mixed disease of ADCI and LBD.

Our first major finding was that when both AD and LBD were considered, LBD, but not AD, was characterized by increased TBR in all lobar regions. This finding is consistent with those of previous studies, which demonstrated that increased theta power and decreased alpha and beta power characterize DLB^17,19^, and are predictive of cognitive deterioration in patients with PD^43^. These EEG findings of generalized slowing have also been reported in patients with AD^44^. However, considering the high prevalence of mixed pathology of AD and LBD in patients with cognitive impairment^7,8^, our findings suggest that the concomitant presence of Lewy body pathology could be associated with previously reported generalized EEG slowing in patients with AD. In addition, sensitivity analyses based on the presence of DLB (rather than LBD) produced similar results. Regardless of the type of LBD (PD or DLB), increased TBR was associated with the presence of LBD. Although the diagnostic performance of TBR in detecting LBD was not high in our study (probably due to the small sample size in the control group), increased central and parietal TBR could be a useful index to identify Lewy body pathology in patients with cognitive impairment. The mechanism underlying the increase in TBR in patients with LBD is unknown. However, previous studies have proposed cholinergic depletion as an underlying mechanism to explain abnormal EEG findings in patients with DLB^45^. The degeneration of the pedunculopontine tegmental nucleus in patients with LBD^46^ and the resultant loss of cholinergic modulation of thalamo-cortical^28^ and thalamo-striatal connections^47^ could be associated with the pathological increase in TBR.

Our second major finding was that the presence of AD and ADCI had different effects on TAR. Specifically, the presence of AD was not independently associated with TAR after controlling for the effect of LBD. However, our sensitivity analyses revealed that the presence of ADCI (amyloid-positivity) and LBD were independently associated with increased frontal, central and occipital TAR and interactively associated with increased occipital TAR. As the ADCI group had TAR comparable with those in the control group in all lobar regions, while the mixed disease group (with ADCI and LBD) had significantly higher TAR (Table 2) than the ADCI group, the effects of ADCI on TAR could be mainly driven by significantly increased TAR in the mixed disease group. By virtue of the combined use of FDG PET and FBB PET, we could differentiate patients having LBD with AD from those having LBD with amyloid deposition based on the presence of entorhinal hypometabolism^40,48^. As the only difference between the original and the sensitivity analysis was that the patients with DLB with amyloid deposition were included in the mixed disease group rather than in the LBD group in the sensitivity analysis, it is suggested that increased TAR could sensitively reflect the brain changes originating from LBD with amyloid deposition. Although further studies correlating imaging biomarkers with QEEG changes are needed, the significant interaction of ADCI and LBD on occipital TAR was reminiscent of the previously reported interaction between dopaminergic depletion and occipital amyloid deposition in LBD patients^49^.

Our third major finding was that increased TBR in all lobar regions and temporal TAR were associated with an increased risk of hallucinations in patients with cognitive impairment. This finding is consistent with those of previous studies, which reported that PD patients with visual hallucinations had significantly higher theta power and lower beta and gamma powers compared with those without visual hallucination^16^. Furthermore, a higher connectivity index in the theta band is associated with more frequent and severe visual hallucinations in patients with DLB^15^. The cholinergic dysfunction that is reflected in increased TBR could be implicated in the pathogenesis of hallucinations^50^. Meanwhile, other core features of LBD including parkinsonism and cognitive fluctuations were not significantly associated with TBR or TAR in our cognitively impaired patients. Our results suggest that the underlying mechanism for hallucinations might be different from cognitive fluctuations or parkinsonism. Considering that multiple brain changes, including dopaminergic depletion, cholinergic degeneration^12,51^, and network derangement^52^, could contribute to clinical symptoms in LBD, further multifactorial imaging studies are needed to reveal the complex mechanisms underlying the QEEG changes and various clinical symptoms in LBD.

Between TBR and TAR in all lobar regions, central and parietal TBR had the highest AUC for identifying LBD. This finding is not consistent with that of a recent QEEG study, wherein TAR best differentiated MCI due to DLB from that due to AD^17^. We performed DAT-PET in all AD patients with significant parkinsonism (UPDRS part III score > 16) and classified them into the mixed disease group based on the presence of significant DAT depletion. Considering that patients in the LBD group had significantly higher TBR than those in the control group in our study, and that previous studies have reported low diagnostic sensitivity of DLB in patients with AD^53^, underestimated concomitant Lewy body pathology in patients with AD could significantly deteriorate the diagnostic performance of TBR to differentiate DLB from AD in the previous study. This point of view is further supported by the association of TBR with frontal/executive dysfunction and hallucinations, which are known characteristics of LBD^54^. Meanwhile, increased TAR was associated with language, memory and visuospatial dysfunction, which are the neuropsychological hallmarks of AD^55^ and DLB^56^, respectively. Our previous study demonstrated that the presence of ADCI and LBD has independent detrimental effects on temporo-parietal cortical thinning and visuospatial dysfunction^8^, and the mixed disease group with ADCI and LBD had increased TAR compared with the control, ADCI, and LBD groups (Table 2). Therefore, increased TAR could reflect the neurodegeneration caused by concomitant ADCI and LBD, which could be further associated with language, memory, and visuospatial dysfunction.

This study had several limitations. First, we could not perform pathologic confirmation or tau PET in this study. However, all our AD and LBD patients satisfied the research criteria for AD and LBD, and the diagnosis of our AD and LBD patients were based on meticulous clinical evaluation as well as amyloid and dopamine transporter PET results. Moreover, we considered the evidence of neuronal injury on FDG PET to diagnose AD patients. Second, we investigated the implications of the ratio between the lobar-averaged band power activities. In addition, we used simple spectral power analysis of EEG acquired from a low-density scalp EEG, this might contribute to the moderate discriminatory ability of central TBR. Although we aimed to identify easily achievable QEEG biomarkers using TAR and TBR in this study, future studies investigating connectivity within certain frequency bands and inter-lead or inter-frequency connectivity with high-density EEG recording are needed. Third, we could not investigate the core clinical features of LBD and perform detailed neuropsychological tests in the control participants, which prevented the consideration of the effect of normal aging in the analyses of clinical symptoms and cognitive dysfunction. Future EEG studies on imaging biomarker-validated control participants are warranted. Forth, the diagnostic performance of TAR and TBR in this study was relatively lower than that reported in previous studies, and this might be due to the small size of the control group. In addition, the diagnostic performance of TAR and TBR in distinguishing between LBD and mixed disease and other causes of cognitive impairment was not determined in this study. Future studies with larger sample sizes and wider spectra of diseases, including frontotemporal dementia, are needed to confirm the discriminating value of TAR and TBR.

## Methods

### Participants

Seventy-four patients with AD and/or LBD were consecutively recruited from 2018 to 2020 at the dementia clinic of Yonsei University Severance Hospital, Seoul, Korea. All patients underwent neurological examination, neuropsychological tests, 3-Tesla MRI, and FDG PET. The clinical features of AD, including slowly progressive memory problems, and those of LBD including parkinsonism, rapid eye movement sleep behavior disorder (RBD), visual hallucinations, and cognitive fluctuation, were evaluated using semi-structured questionnaires administered to caregivers. The severity of Parkinsonism was assessed based on the Movement Disorder Society UPDRS part III score and was considered moderate if the score was > 16.

All patients with AD fulfilled the criteria for probable AD dementia with high levels of biomarker evidence^57^, and all patients with MCI due to AD met the criteria for a high likelihood of MCI due to AD from the National Institute on Aging-Alzheimer’s Association workgroups guidelines for AD^58^. All patients were identified as having evidence of neuronal injury on FDG PET^40,48,59^ and significant cerebral β-amyloid deposition on^18^F-Florbetaben (FBB) PET^60^. Patients with LBD included those with PD and DLB who were recruited using the United Kingdom PD Brain Bank diagnostic criteria^61^ and the 2017 revised criteria for DLB^38^, respectively. PD MCI and PD dementia were diagnosed based on the level II PD-MCI criteria^62^ and the clinical criteria of probable PD dementia^63^, respectively. All patients with MCI due to DLB met the probable DLB criteria except for the presence of dementia. Forty-two out of 58 patients with LBD were confirmed to have dopaminergic depletion on DAT PET using^18^F-N-fluoropropyl-2b-carbomethoxy-3b-(4-iodophenyl) nortropane.

Patients with positive FBB PET scan were considered to have ADCI regardless of cognitive symptoms or FDG PET patterns. Patients with AD dementia and those with MCI due to AD had memory problems as their chief complaint, entorhinal hypometabolism on FDG PET^59^, and positive FBB PET scan. They were regarded as having “typical AD”^40^, which is a more advanced stage of ADCI. Patients who were positive for FBB PET scan but did not have memory problems as their chief complaint or entorhinal hypometabolism were considered as having ADCI but not typical AD. Among the patients with LBD, there were 44 patients with DLB, and 14 patients with PD. As there was no patient with PD with β-amyloid deposition, three categories comprised mixed disease including (1) typical AD with PD, (2) typical AD with DLB, and (3) DLB with β-amyloid deposition. The exclusion criteria were pure vascular cognitive impairment, other causes of degenerative dementia, including frontotemporal dementia, corticobasal degeneration, progressive supranuclear palsy, and other causes of adequate cognitive impairment, including epilepsy, psychiatric disorders, normal-pressure hydrocephalus, drug-induced cognitive impairment, and structural brain lesions (e.g., tumor or hemorrhage).

The control participants did not have any symptoms of cognitive impairment or a history of neurological or psychiatric illnesses. All 17 controls had normal cognitive function as assessed using the Korean version of the Mini-Mental State Examination (K-MMSE). We investigated the medication use in all participants, including acetylcholinesterase inhibitors, antidepressants, antipsychotics, benzodiazepines and dopaminergic medications.

This study was approved by Yonsei University Severance Hospital institutional review board (No. 4-2018-0546). All procedures performed in human studies were in accordance with the ethical standards of the institutional and/or national research committee and with the 1964 Helsinki Declaration and its later amendments or comparable ethical standards. Informed consent was obtained from all participants.

### Neuropsychological test

All patients in the disease groups underwent detailed neuropsychological tests using the Seoul Neuropsychological Screening Battery^64^. Standardized z-scores were available for all scorable tests based on age- and education-matched norms. Among the scorable tests, we included the digit span backward test for the attention domain, the Korean version of the Boston Naming Test for the language domain, the copying item of the Rey–Osterrieth Complex Figure Test (RCFT) for the visuospatial domain, the 20-min delayed recall item of the RCFT and Seoul Verbal Learning Test for the memory domain, and the phonemic Controlled Oral Word Association Test (COWAT), semantic COWAT, and the Stroop color reading test for the frontal/executive domain. Age- and education-matched z-scores were averaged to calculate the scores of each domain. The K-MMSE and CDR-SOB were used to evaluate general cognition.

### EEG acquisition and analysis

All participants underwent EEG using the international 10–20 system for electrode placement. Nineteen channels were used, including FP1, FP2, F7, F3, Fz, F4, F8, T3, C3, Cz, C4, T4, T5, P3, Pz, P4, T6, O1, and O2, with referential montage. The reference electrodes were placed at the bilateral earlobes (A1, A2) and ground electrode was placed on the forehead. The contact impedance was kept below 10 kΩ during the recording. The sampling rate was 200 Hz. Participants were relaxed and awake, with eyes closed during the recording, and resting state EEG data were recorded for at least 5 min. We selected 3 min of eye-closed and artifact-free data based on visual inspection for further analysis. One epoch was 4 s long, and an average of 45 epochs were analyzed. The EEG data were passed through a notch filter. Thereafter, EEG data were filtered with high-pass offline above 1 Hz, and then low-pass filter below 45.5 Hz. The EEG data were recomputed to obtain the common average reference. Artifacts were removed in two steps. The first step was the rejection of non-stationary bad epochs, and the second step was to remove stationary bad components related to electromyogram, electrooculogram, cardiac signals such as heartbeat and slow drift wave such as drowsiness were removed to yield cleaned QEEG data by adaptive mixture independent component analysis (amICA)^65^. At the sensor level, the absolute and relative power of EEG data was calculated in the following eight spectral bands using Welch-based discrete Fourier transformation. We used built-in function in MATLAB for performing Welch method^66^ (window size: sampling rate * 4 s; overlap: sampling rate * 2 s; window type: hamming window): delta (1–4 Hz), theta (4–8 Hz), alpha1 (8–10 Hz), alpha2 (10–12 Hz), beta1 (12–15 Hz), beta2 (15–20 Hz), beta3 (20–30 Hz), and gamma (30–45 Hz). We calculated the TAR and TBR, which are the values obtained after dividing the theta band power density by the alpha or beta band power density for each channel. The mean lobar TAR and TBR were defined as the summation of the corresponding channels in each lobe: FP1, FP2, F3, F4, F7, F8, and Fz leads for the frontal lobe; C3, C4, and Cz leads for the central lobe; T3, T4, T5, and T6 leads for the temporal lobe; P3, P4, and Pz leads for the parietal lobe; and O1 and O2 leads for the occipital lobe. In addition, the EEG relative power density in all frequency bands in all lobar regions were calculated. Logarithmic transformation was applied considering the right-skewed distribution of the TAR and TBR. All EEG preprocessing processes, sensor-level data, source-level data calculation and extraction were performed using a cloud-based AI-driven auto-analyzing platform (iSyncBrain™, iMediSync, Inc.; https://isyncbrain.com).

### Statistical analysis

Statistical analyses for demographic and clinical data were performed using the Statistical Package for the Social Sciences version 25.0 (IBM Corp., Armonk, NY). Receiver operating characteristic (ROC) analyses were performed using R (version 4.0.2; R Foundation for Statistical Computing, Vienna, Austria). Analyses of variance and χ2 tests were used to compare clinical features between the disease and control groups.

Group-wise comparisons of mean lobar TAR, TBR, and log-transformed relative power in each frequency band were performed using GLM after controlling for age, sex, and education. Since there were significant group differences in the frequency of antidepressants and acetylcholinesterase inhibitors use, they were further entered as covariates.

The independent and interaction effects of typical AD and LBD on log-transformed relative power in each frequency band, mean lobar TAR, and mean lobar TBR were also investigated using GLM after controlling for the same covariates. If the interaction terms of typical AD and LBD were significant (p < 0.05), typical AD, LBD, and their interaction terms were simultaneously entered as predictors. Only typical AD and LBD were entered as predictors in cases where their interaction term was not significant. As previous studies have shown that patients with PD and DLB have more prominent QEEG changes than AD patients, we categorized our study participants according to the presence of typical AD and the presence of LBD (PD + DLB). However, to determine the effects of different classifications based on the presence of ADCI (rather than typical AD) or that based on the presence of DLB (rather than LBD), further GLMs were performed as sensitivity analyses.

To determine the association of mean lobar TAR and TBR with core clinical features of LBD, we performed logistic regression analyses for the presence of visual hallucinations and cognitive fluctuations in patients with cognitive impairment after controlling for age, sex, education, disease duration, and the use of antidepressants and acetylcholinesterase inhibitors. To evaluate the association of mean TAR and TBR with the severity of parkinsonism, GLMs for the UPDRS part III score were performed after controlling for the same covariates with logistic regression analyses. To evaluate the association between EEG biomarkers and cognitive dysfunction, GLMs for neuropsychological test scores were performed after controlling for sex, disease duration, and the use of antidepressants and acetylcholinesterase inhibitors since neuropsychological test scores were age- and education-matched z-scores. We applied the FDR method to correct multiple comparisons, and FDR-corrected p-values < 0.05 were considered to be statistically significant.

The diagnostic accuracy based on lobar TAR or lobar TBR was evaluated by calculating the area under the receiver operating characteristic curve (AUC), with 95% confidence intervals (CIs). AUCs were compared using the DeLong method.

### Ethics approval

Institutional Review Board of the Yonsei University Severance Hospital.

## Supplementary Information

Supplementary Information.

## Data Availability

The data and codes used in this study are available from the corresponding authors upon reasonable request.
