# Supplementary material for: Implication of EEG theta/alpha and theta/beta ratio in Alzheimer’s and Lewy body disease
Source: Sci Rep. 2022 Nov 4;12:18706. doi: 10.1038/s41598-022-21951-5 (PMC9636216; doi:10.1038/s41598-022-21951-5)
Supplement: Supplementary file 1 — Supplementary Information. [file 41598_2022_21951_MOESM1_ESM.docx]

**Supplementary Tables
Supplementary table 1.** Group-wise comparisons of mean lobar relative band power

|  | Control | AD | Mixed disease | LBD | P value |
| --- | --- | --- | --- | --- | --- |
| Delta |  |  |  |  |  |
| Frontal | -1.70 (0.41) | -1.45 (0.38) | -1.26 (0.42) | -1.36 (0.53) | 0.200 |
| Central | -1.90 (0.50) | -1.62 (0.36) | -1.46 (0.38) | -1.52 (0.55) | 0.197 |
| Temporal | -1.86 (0.40) | -1.58 (0.43) | -1.37 (0.42) | -1.54 (0.58) | 0.197 |
| Parietal | -1.93 (0.39) | -1.72 (0.43) | -1.46 (0.49) | -1.55 (0.60) | 0.210 |
| Occipital | -2.31 (0.65) | -1.92 (0.69) | -1.62 (0.52) | -1.80 (0.73) | 0.197 |
| Theta |  |  |  |  |  |
| Frontal | -1.96 (0.40) | -1.63 (0.36)^a,b^ | -1.30 (0.35)^a,b,d^ | -1.60 (0.53)^a,d^ | 0.004 |
| Central | -2.09 (0.35) | -1.69 (0.37)^a,b^ | -1.33 (0.35)^a,b^ | -1.55 (0.51)^a^ | < 0.001 |
| Temporal | -1.94 (0.40) | -1.62 (0.38)^a,b^ | -1.28 (0.39)^a,b^ | -1.49 (0.59)^a^ | 0.008 |
| Parietal | -2.06 (0.30) | -1.79 (0.37)^a,b^ | -1.40 (0.39)^a,b^ | -1.63 (0.53)^a^ | 0.004 |
| Occipital | -2.16 (0.58) | -1.76 (0.50)^a^ | -1.37 (0.41)^a^ | -1.63 (0.65)^a^ | 0.006 |
| Alpha1 |  |  |  |  |  |
| Frontal | -1.26 (0.50) | -1.75 (0.64)^a^ | -2.43 (0.57)^a^ | -1.96 (0.73)^a^ | 0.017 |
| Central | -1.32 (0.52) | -1.74 (0.64) | -2.15 (0.56) | -1.89 (0.61) | 0.056 |
| Temporal | -1.14 (0.34) | -1.50 (0.50)^a,b^ | -2.04 (0.61)^a,b^ | -1.83 (0.66)^a^ | 0.009 |
| Parietal | -1.34 (0.47) | -1.71 (0.66) | -2.04 (0.56) | -1.87 (0.61) | 0.197 |
| Occipital | -0.82 (0.42) | -1.28 (0.67)^b^ | -1.91 (0.74)^a,b^ | -1.53 (0.79)^a^ | 0.010 |
| Alpha2 |  |  |  |  |  |
| Frontal | -2.58 (0.53) | -2.58 (0.43) | -2.90 (0.52) | -2.77 (0.74) | 0.264 |
| Central | -2.53 (0.51) | -2.57 (0.32) | -2.74 (0.43) | -2.60 (0.68) | 0.457 |
| Temporal | -2.43 (0.46) | -2.52 (0.32) | -2.84 (0.56) | -2.72 (0.79) | 0.197 |
| Parietal | -2.21 (0.54) | -2.26 (0.44) | -2.63 (0.50) | -2.44 (0.73) | 0.197 |
| Occipital | -2.46 (0.71) | -2.35 (0.58) | -2.56 (0.67) | -2.58 (0.89) | 0.618 |
| Beta1 |  |  |  |  |  |
| Frontal | -3.09 (0.40) | -3.01 (0.39) | -3.17 (0.68) | -3.21 (0.52) | 0.604 |
| Central | -2.86 (0.40) | -2.79 (0.39) | -2.98 (0.56) | -2.98 (0.46) | 0.464 |
| Temporal | -2.91 (0.32) | -2.96 (0.44) | -3.16 (0.64) | -3.16 (0.56) | 0.360 |
| Parietal | -2.68 (0.45) | -2.72 (0.41) | -2.86 (0.63) | -2.92 (0.53) | 0.379 |
| Occipital | -3.38 (0.48) | -3.10 (0.50) | -3.00 (0.82) | -3.25 (0.56) | 0.565 |
| Beta2 |  |  |  |  |  |
| Frontal | -2.61 (0.33) | -2.80 (0.48) | -3.03 (0.72) | -3.02 (0.65) | 0.267 |
| Central | -2.45 (0.37) | -2.58 (0.44) | -2.79 (0.67) | -2.83 (0.59) | 0.232 |
| Temporal | -2.62 (0.32) | -2.82 (0.52) | -3.05 (0.68) | -3.07 (0.68) | 0.197 |
| Parietal | -2.34 (0.30) | -2.55 (0.54) | -2.82 (0.68) | -2.84 (0.67) | 0.197 |
| Occipital | -3.00 (0.42) | -3.03 (0.52) | -3.09 (0.83) | -3.23 (0.65) | 0.692 |
| Beta3 |  |  |  |  |  |
| Frontal | -2.53 (0.52) | -2.63 (0.70) | -2.95 (0.75) | -2.93 (0.82) | 0.360 |
| Central | -2.31 (0.62) | -2.35 (0.59) | -2.68 (0.80) | -2.75 (0.74) | 0.197 |
| Temporal | -2.73 (0.43) | -2.81 (0.70) | -3.05 (0.70) | -3.11 (0.79) | 0.264 |
| Parietal | -2.46 (0.52) | -2.46 (0.65) | -2.88 (0.75) | -2.91 (0.78) | 0.197 |
| Occipital | -3.18 (0.58) | -3.11 (0.71) | -3.16 (0.84) | -3.37 (0.82) | 0.662 |

Data are expressed in mean (standard deviation). P values are results of general linear models for mean lobar relative power in each frequency band using the disease group as a predictor after controlling for age, sex, education, use of AchEI and antidepressants.

^a^ Significantly different in the comparison with the control group.

^b^ Significantly different in the comparison between the AD and Mixed disease groups.

^c^ Significantly different in the comparison between the AD and LBD groups.

^d^ Significantly different in the comparison between the mixed disease and LBD groups.

Abbreviations: AChEI, acetylcholinesterase inhibitor; AD, Alzheimer’s disease; LBD, Lewy body disease.

**Supplementary table 2.** Independent effects of AD and LBD on lobar relative band power

|  | LBD effect |  | AD effect |  |
| --- | --- | --- | --- | --- |
|  | Beta (SE) | FDR | Beta (SE) | FDR |
| Delta |  |  |  |  |
| Frontal | 0.24 (0.11) | 0.065 | 0.15 (0.10) | 0.303 |
| Central | 0.25 (0.12) | 0.064 | 0.17 (0.11) | 0.289 |
| Temporal | 0.22(0.12) | 0.085 | 0.22 (0.11) | 0.183 |
| Parietal | 0.26 (0.12) | 0.065 | 0.16 (0.11) | 0.303 |
| Occipital | 0.35 (0.17) | 0.065 | 0.25 (0.15) | 0.285 |
| Theta |  |  |  |  |
| Frontal | 0.41 (0.11) | 0.002 | 0.33 (0.10) | 0.035 |
| Central | 0.50 (0.11) | 0.00028 | 0.31 (0.10) | 0.035 |
| Temporal | 0.44 (0.12) | 0.002 | 0.25 (0.11) | 0.100 |
| Parietal | 0.46 (0.11) | 0.001 | 0.26 (0.10) | 0.088 |
| Occipital | 0.51 (0.14) | 0.002 | 0.34 (0.12) | 0.223 |
| Alpha1 |  |  |  |  |
| Frontal | -0.49 (0.15) | 0.009 | -0.35 (0.14) | 0.100 |
| Central | -0.37 (0.14) | 0.039 | -0.31 (0.13) | 0.100 |
| Temporal | -0.50 (0.13) | 0.002 | -0.27 (0.12) | 0.131 |
| Parietal | -0.31 (0.14) | 0.064 | -0.24 (0.13) | 0.223 |
| Occipital | -0.61 (0.17) | 0.005 | -0.42 (0.16) | 0.088 |
| Alpha2 |  |  |  |  |
| Frontal | -0.31 (0.15) | 0.065 | -0.17 (0.14) | 0.371 |
| Central | -0.16 (0.13) | 0.247 | -0.18 (0.12) | 0.293 |
| Temporal | -0.36 (0.15) | 0.054 | -0.19 (0.14) | 0.326 |
| Parietal | -0.34 (0.15) | 0.061 | -0.22 (0.14) | 0.285 |
| Occipital | -0.25 (0.18) | 0.193 | -0.08 (0.17) | 0.854 |
| Beta1 |  |  |  |  |
| Frontal | -0.18 (0.13) | 0.193 | 0.02 (0.12) | 0.936 |
| Central | -0.20 (0.12) | 0.116 | -0.01 (0.11) | 0.936 |
| Temporal | -0.25 (0.13) | 0.078 | -0.05 (0.12) | 0.854 |
| Parietal | -0.25 (0.13) | 0.082 | -0.01 (0.12) | 0.936 |
| Occipital | 0.06 (0.15) | 0.693 | 0.22 (0.14) | 0.289 |
| Beta2 |  |  |  |  |
| Frontal | -0.31 (0.15) | 0.066 | -0.09 (0.14) | 0.777 |
| Central | -0.31 (0.14) | 0.061 | -0.06 (0.13) | 0.854 |
| Temporal | -0.34 (0.15) | 0.061 | -0.09 (0.14) | 0.777 |
| Parietal | -0.37 (0.15) | 0.051 | -0.09 (0.14) | 0.777 |
| Occipital | -0.11 (0.16) | 0.533 | 0.06 (0.15) | 0.854 |
| Beta3 |  |  |  |  |
| Frontal | -0.35 (0.18) | 0.078 | -0.08 (0.17) | 0.854 |
| Central | -0.42 (0.18) | 0.054 | -0.03 (0.16) | 0.936 |
| Temporal | -0.35 (0.17) | 0.065 | -0.02 (0.16) | 0.936 |
| Parietal | -0.45 (0.17) | 0.042 | -0.02 (0.16) | 0.936 |
| Occipital | -0.10 (0.19) | 0.602 | 0.12 (0.17) | 0.777 |

Data are results of general linear models for mean lobar relative band power in each frequency band using the presence of AD and LBD as predictors after controlling for age, sex, education, use of AchEI antidepressants.

Abbreviations: AChEI, acetylcholinesterase inhibitor; AD, Alzheimer’s disease; LBD, Lewy body disease; SE, standard error.

**Supplementary Table 3.** Group-wise comparisons of mean lobar TAR and TBR based on the presence of AD and DLB

|  | Control | AD | Mixed disease | DLB | P value |
| --- | --- | --- | --- | --- | --- |
| TAR |  |  |  |  |  |
| Frontal TAR | -0.20 (0.85) | -0.05 (0.68) | 0.57 (0.66) | -0.04 (0.97) | 0.141 |
| Central TAR | -0.39 (0.80) | -0.17 (0.64) | 0.44 (0.66) | -0.08 (0.88) | 0.117 |
| Temporal TAR | -0.22 (0.85) | -0.20 (0.66) | 0.53 (0.72) | 0.01 (1.01) | 0.141 |
| Parietal TAR | -0.50 (0.67) | -0.36 (0.68) | 0.24 (0.68) | -0.23 (0.88) | 0.141 |
| Occipital TAR | -0.40 (1.29) | -0.61 (0.86) | 0.18 (0.90) | -0.47 (1.11) | 0.174 |
| TBR |  |  |  |  |  |
| Frontal TBR | 0.23 (0.50) | 0.77 (0.77)^a,b^ | 1.35 (0.93)^a,b^ | 0.92 (0.99)^a^ | 0.015 |
| Central TBR | -0.02 (0.54) | 0.52 (0.73)^a,b^ | 1.10 (0.97)^a,b^ | 0.78 (0.93)^a^ | 0.010 |
| Temporal TBR | 0.24 (0.59) | 0.81 (0.91)^a^ | 1.40 (0.94)^a^ | 1.07 (1.12)^a^ | 0.015 |
| Parietal TBR | -0.14 (0.42) | 0.38 (0.81)^a,b^ | 1.05 (0.93)^a,b^ | 0.70 (1.03)^a^ | 0.010 |
| Occipital TBR | 0.41 (0.73) | 0.76 (0.87) | 1.33 (1.09) | 1.07 (1.07) | 0.054 |

Data are expressed in mean (standard deviation). P values are results of general linear models for TAR and TBR using the disease group as a predictor after controlling for age, sex, education, use of AchEI and antidepressants. P values are FDR corrected.

^a^ Significantly different in the comparison with the control group.

^b^ Significantly different in the comparison between the AD and Mixed disease groups.

^c^ Significantly different in the comparison between the AD and DLB groups.

^d^ Significantly different in the comparison between the mixed disease and DLB groups.

Abbreviations: AChEI, acetylcholinesterase inhibitor; AD, Alzheimer’s disease; DLB, Dementia with Lewy bodies; FDR, False discovery rate; TAR, log transformed theta/lpha ratio; TBR, log transformed theta/beta ratio.

**Supplementary Table 4.** Independent effects of AD and DLB on lobar TAR and TBR

|  | DLB effect |  | AD effect |  |
| --- | --- | --- | --- | --- |
|  | Beta (SE) | P value | Beta (SE) | P value |
| TAR |  |  |  |  |
| Frontal TAR | 0.38 (0.22) | 0.099 | 0.42 (0.21) | 0.073 |
| Central TAR | 0.43 (0.20) | 0.058 | 0.42 (0.19) | 0.056 |
| Temporal TAR | 0.43 (0.22) | 0.068 | 0.30 (0.21) | 0.172 |
| Parietal TAR | 0.39 (0.20) | 0.068 | 0.35 (0.19) | 0.091 |
| Occipital TAR | 0.35 (0.28) | 0.215 | 0.29 (0.26) | 0.262 |
| TBR |  |  |  |  |
| Frontal TBR | 0.76 (0.22) | 0.003 | 0.57 (0.21) | 0.050 |
| Central TBR | 0.83 (0.22) | 0.001 | 0.54 (0.20) | 0.050 |
| Temporal TBR | 0.85 (0.24) | 0.003 | 0.53 (0.23) | 0.055 |
| Parietal TBR | 0.88 (0.23) | 0.001 | 0.52 (0.22) | 0.055 |
| Occipital TBR | 0.75 (0.24) | 0.006 | 0.42 (0.23) | 0.091 |

Data are results of general linear models for TAR and TBR using the presence of DLB and ADD as predictors after controlling for age, sex, education, use of AchEI and antidepressants. P values are FDR corrected.

Abbreviations: AChEI, acetylcholinesterase inhibitor; AD, Alzheimer’s disease; DLB, Dementia with Lewy bodies; FDR, False discovery rate; SE, standard error; TAR, log transformed theta/alpha ratio; TBR, log transformed theta/beta ratio.

**Supplementary table 5.** Association of TAR and TBR with UPDRS part III

|  | UPDRS part III |  |
| --- | --- | --- |
|  | Beta (SE) | P value |
| TAR |  |  |
| Frontal TAR | 2.20 (1.91) | 0.255 |
| Central TAR | 2.68 (2.16) | 0.255 |
| Temporal TAR | 3.39 (1.91) | 0.135 |
| Parietal TAR | 2.51 (2.10) | 0.255 |
| Occipital TAR | 2.82 (1.68) | 0.141 |
| TBR |  |  |
| Frontal TBR | 3.36 (1.68) | 0.116 |
| Central TAR | 4.02 (1.73) | 0.093 |
| Temporal TBR | 2.97 (1.53) | 0.116 |
| Parietal TBR | 3.73 (1.58) | 0.093 |
| Occipital TBR | 3.48 (1.55) | 0.093 |

Data are results of general linear models for UPDRS part III performed in cognitively impaired patients age, sex, education, disease duration, use of AchEI and antidepressants. P values are FDR corrected.

Abbreviations: AChEI, acetylcholinesterase inhibitor; FDR, False discovery rate; TAR, log transformed theta/alpha ratio; TBR, log transformed theta/beta ratio; SE, standard error; UPDRS, Unified Parkinson’s Disease Rating Scale.
